# Isolation and characterization of bovine parainfluenza virus type 3 from water buffaloes (*Bubalus bubalis*) in Argentina

**DOI:** 10.1186/1746-6148-8-83

**Published:** 2012-06-20

**Authors:** Silvina S Maidana, Patricia M Lomonaco, Gustavo Combessies, María I Craig, Julian Diodati, Daniela Rodriguez, Viviana Parreño, Osvaldo Zabal, José L Konrad, Gustavo Crudelli, Axel Mauroy, Etienne Thiry, Sonia A Romera

**Affiliations:** 1 Instituto de Virología, Centro de Investigaciones en Ciencias veterinarias y Agronómicas (CICVyA), Instituto de tecnología Agropecuaria (INTA), N. Repetto y Los Reseros S/N, CC25 (B1712WAA), Buenos Aires, Castelar, Argentina; 2 Facultad de Veterinaria, Universidad Nacional del Nordeste, 25 de Mayo 868, Corrientes 3400, Argentina; 3 Consejo Nacional de Investigaciones Científicas y Tecnológicas (CONICET), Rivadavia 1917 (C1033AAJ), Ciudad Autónoma de Buenos Aires, Argentina; 4 Veterinary Virology and Animal Viral Diseases, Department of Infectious and Parasitic Diseases, Faculty of Veterinary Medicine, University of Liège, Boulevard de Colonster, 20, B43b, Liège, B-4000, Belgium; 5 Laboratorio Azul, Av., 25 de Mayo 479/485 (7300), Azul, Buenos Aires, Argentina

## Abstract

**Background:**

Parainfluenza virus type 3 (PIV3) was isolated from dairy buffaloes (*Bubalus bubalis*) naturally affected with respiratory and reproductive clinical conditions.

**Results:**

Examination of nasal and vaginal swabs collected from 12 diseased buffaloes led to the isolation of three paramyxovirus isolates from two animals. Antigenic, morphological and biological characteristics of these three isolates were essentially similar to those of members of the *Paramyxoviridae* family. Antigenic analysis by direct immunofluorescence and cross neutralization test placed these isolates together with bovine parainfluenza virus type 3 (BPIV3). Nucleotide and amino acid phylogenetic analysis of partial matrix gene sequences of the buffalo isolates and six field BPIV3 isolates from bovines in Argentina were studied. Buffalo isolates were similar to genotype B (BPIV3b) while the six BPIV3 isolates were similar to genotypes A (BPIV3a) and C (BPIV3c).

**Conclusions:**

This is the first characterization of BPIV3 in water buffalo.

According to the samples analyzed, in Argentina, the genotype B was found in buffalo and the genotypes A and C were found in cattle.

## Background

Paramyxoviruses are well-known pathogens of the central nervous and respiratory systems of many host species. In the last few decades, many novel paramyxoviruses have emerged causing devastating illnesses in different aquatic and terrestrial animals, including in some cases a species jump to humans [1]. Parainfluenza virus type 3 (PIV3) is an enveloped, single-stranded negative sense RNA virus within the *Respirovirus* genus of the *Paramyxoviridae* family [2,3]. The *Respirovirus* genus includes human parainfluenza virus types 1 and 3 (HPIV1 and HPIV3, respectively), Sendai virus (murine PIV1) and bovine parainfluenza virus type 3 (BPIV3) [2]. In some instances when animals are subjected to high stressful conditions, infection with BPIV3 can contribute to tissue damage and immunosuppression, resulting in severe bronchopneumonia from secondary bacterial infections [4]. The resulting disease is part of the bovine respiratory disease complex (BRDC) and is considered as the most significant illness associated with feedlot cattle in the USA [5], and possibly worldwide. Other respiratory viruses such as bovine herpesvirus 1, bovine viral diarrhea virus (BVDV) and bovine respiratory syncytial virus (BRSV) have also been associated with BRDC development in feedlot cattle [6]. The clinical presentation of bovine infections with BPIV3 can vary considerably, ranging from asymptomatic infections to severe respiratory illness. In most cases where BPIV3 is implicated in disease, usual clinical signs include coughing, fever and nasal discharge [6].

Three genotypes, A (BPIV3a), B (BPIV3b) and C (BPIV3c) have been described, based on genetic and phylogenetic analysis [7]. The BPIV3b genotype could hypothetically be a lineage from a strain that recently crossed from another host species into cattle [8]. PIV3 infections were found in a wide variety of mammals including cattle, humans, sheep [9], goats [10], bison [11], guinea pigs [12], black and white rhinoceros [13], moose [14], bighorn sheep [15] and camels [16]. Cross-species infections have been reported in numerous instances, including HPIV-3 in guinea pigs [12], BPIV3 in a human, BPIV3 in sheep, and ovine PIV3 in cattle [17]. In Argentina, serological studies conducted in 1980 and 1984 showed a high incidence of antibodies against BPIV3 (77%) in cattle from the main livestock breeding regions [18,19]. In addition, positive serology was reported in domestic and wild South American camelids [20-22] and the virus was isolated from cattle and sheep [23]. However, little is known about the circulation of BPIV3 in cattle and other ruminants in Argentina, as well as about the genotypes that are present in this Southern American country.

Production systems have evolved to mixed managements, where alternative production species, such as the water buffalo (*Bubalus bubalis*), coexist in the same habitat as with cattle. Water buffalo breeding represents an important economic alternative in Argentina, which allows access to national and international markets. This species is susceptible to several viral infections [20,24,25] including BPIV3 as reported in a very early in 1966 in Egypt [26]. In addition to its potential relevance with respect to water buffalo health, this finding bears epidemiological significance due to the risk of transmission of the virus to cattle. The aim of this study was to characterize antigenically and genetically BPIV3 isolates from a respiratory and reproductive syndrome outbreak in dairy buffaloes and compare the buffalo isolate with contemporaneous BPIV3 circulating in cattle.

## Results

### Morphological and biological properties of buffalo viral isolates

PIV3 was successfully isolated from two out of twelve water buffaloes studied, in one case from both nasal (7 N) and vaginal (7 V) swabs, and in the other only from the vaginal swab (2 V).

**Figure 1:**
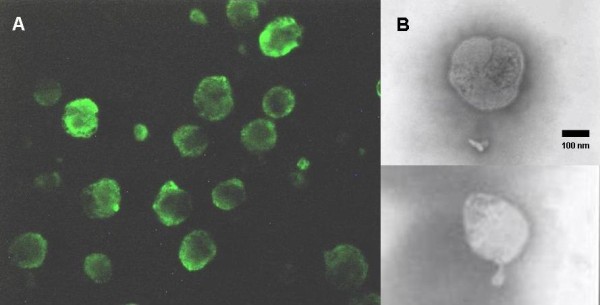
** Microscopic examination of PIV3 isolated from buffaloes. A**: Positive MDBK cells by IF, as observed by epifluorescence in an Olympus BX 40 + H hal microscope (400X magnification). **B**: Electron micrograph of two purified virions of the 7 N isolate, morphologically indistinguishable from paramixoviruses.

Isolates produced characteristic cytopathic effects (CPE) on MDBK cells, similar to those of the BPIV3 reference strain, with many scattered, rounded, refractory cells and small syncytia. CPE were first visualized on the second passage, after three days post inoculation (DPI), and CPE were observed at day 4 or 5 DPI. Both bovine polyclonal anti-BPIV3 serum and FITC-anti-BPIV3 IgG reacted with the cells infected by these isolates (Figure 1 A).

Transmission electron microscopy revealed spherical to pleomorphic virions, approximately 100–300 nm in diameter, which were morphologically indistinguishable from paramyxoviruses. Intact virions were enveloped and densely packed, as has been described for BPIV3. Nucleocapsids were visible and exhibited a typical “herringbone” pattern (Figure 1 B).

The three isolates (7 N, 7 V and 2 V) were able to agglutinate red blood cells (RBCs), with UHA titers of 16, 8 and 4 respectively. The agglutinated RBCs eluted after 1 h suggesting neuraminidase function/activity. The HI test showed 58% of positive reactions, with titers ranging between 1.6 and 2.5 (data not shown).

### Molecular characterization RT-PCR and sequencing

**Figure 2:**
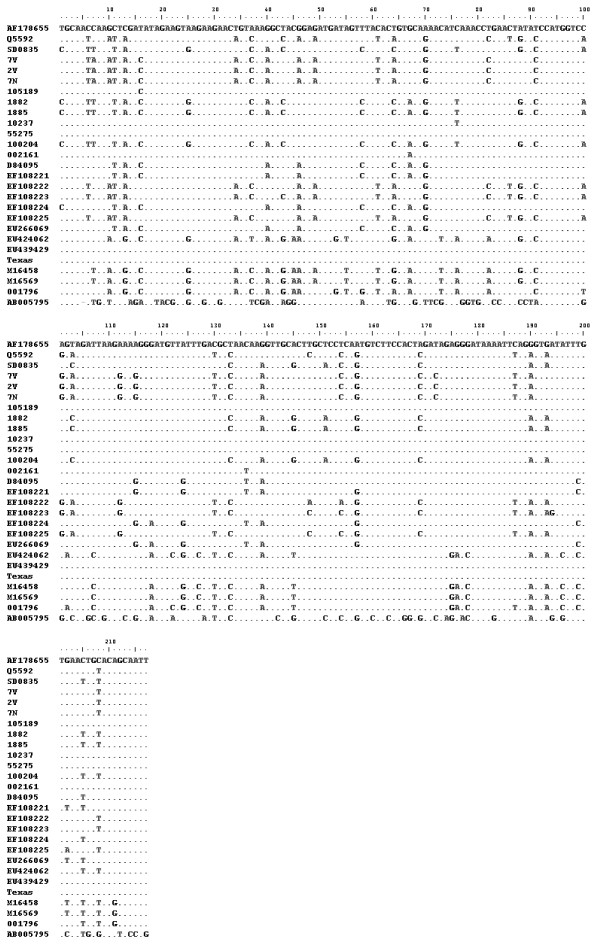
** Nucleotide alignment of M fragment sequences of Argentine buffalo and bovine isolates, other bovine parainfluenza virus 3 and human parainfluenza virus 3 that circulate in the world.** Dots indicate identity with respect to SF strain (AF178655).

A fragment of the M gene, consistent with the expected size of 328 bp was amplified from the 3 virus isolates (7 N, 7 V and 2 V) by RT-PCR. As expected, the primer pairs did not amplify using genomic material of BRSV as template. Analysis of the alignments of 271 pb of the corresponding sequences revealed that they are related to BPIV3 strain Q5592 from Australia with 94% nucleotide identity (Figure 2) and 100% predicted amino acid identity (Table 1).

**Table 1:** Comparison of identity percentages in amino acid and nucleotide sequences of the 217-bp region of the M gene among buffalo and bovine isolates and reference strains of BPIV3

	**BPIV3a ref strain**	**BPIV3b ref strain**	**BPIV3c ref strain**	**Buffalo isolates**			**Bovine isolates**						**Sendai virus**	**Amino acid identity**
				**BPIV3b**			**BPIV3c**			**BPIV3a**				
	AF178655	Q5592	SD0835	7 V	2 V	7 N	1882	1885	100204	105189	55275	10237	AB005795	
AF178655	ID	97	99	97	97	97	99	99	99	100	100	100	65	
Q5592	87	ID	99	100	100	100	99	99	99	97	97	97	65	
SD0835	87	89	ID	99	99	99	100	100	100	99	99	99	64	
7 V	86	94	88	ID	100	100	99	99	99	97	97	97	65	
2 V	86	94	88	100	ID	100	99	99	99	97	97	97	65	
7 N	86	94	88	100	100	ID	99	99	99	97	97	97	65	
1882	87	88	99	87	87	87	ID	100	100	99	99	99	64	
1885	87	88	99	87	87	87	100	ID	100	99	99	99	64	
100204	87	88	99	87	87	87	100	100	ID	99	99	99	64	
105189	100	86	87	87	87	87	87	87	87	ID	100	100	65	
55275	100	87	87	86	86	86	87	87	87	100	ID	100	65	
10237	100	86	88	86	86	86	87	87	87	99	100	ID	65	
AB005795	64	61	65	63	63	63	64	64	64	64	64	63	ID	
**Nucleotide identity**														

*ID*: identical; *ref*: reference.

### Data and phylogenetic analysis

**Figure 3:**
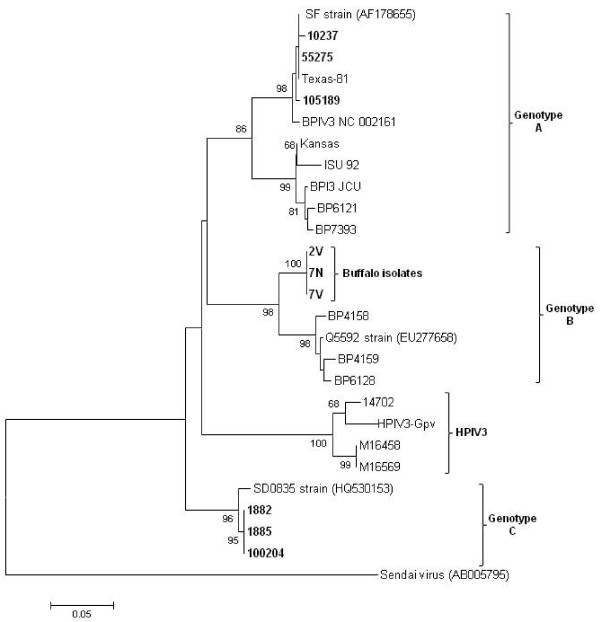
**Phylogenetic analysis based on M gene fragment.** Phylogenetic analysis was performed using MEGA 4 software with 1,000 bootstrap replicates. Phylogenetic analysis based on M gene fragment nucleotide sequences compared to other *Respiroviru*s genus members. The Argentinean isolates are indicated in bold. GenBank accession numbers are presented in Materials and Methods.

Phylogenetic analysis of the M gene fragment of the three Argentinean buffalo isolates (7 N, 7 V and 2 V) together with other *paramyxoviruses*, placed the buffalo isolates in the same clade as BPIV3 (Figure 3).

In particular, the isolates grouped with the BPIV3b genotype represented in the tree by an Australian isolate (Q5592). BPIV3b is distinct from the previously characterized members of BPIV3 represented by genotypes A and C.

In order to determine whether the PIV3 genotype found in buffalo is also circulating in cattle, the same gene fragment was amplified by RT-PCR using as template BPIV3 RNA isolated from six bovines of the Buenos Aires region. Three of the six isolates show 98% nucleotide identity and 100% amino acid identity with BPIV3c (SD0835 strain) reference isolate, and the remaining three, 99% nucleotide identity and 100% amino acid identity with BPIV3a (SF strain) reference isolate (Table 1). Phylogenetic characterization showed that the bovine isolates belong to the BPIV3a and BPIV3c genotypes, which have been reported so far in North America, Europe and China (Figure 3).

### Serum neutralization test

Buffalo sera (n = 24) were analyzed by cross-neutralization, Three were negative for both viruses, BPIV3a and b, 9 showed higher titers against BPIV3b isolated from buffalo, 4 showed higher titers against BPIV3a and the remaining 11 had same titers for both viruses. (Table 2). In general, sera from buffaloes showed higher antibody titers against the isolated buffalo virus than against the reference virus.

**Table 2:** Comparison of neutralizing titers against BPIV3b isolated from buffaloes and against a BPIV3a reference strain

**24 buffaloes**		*Antibody titers against BPIV3b isolate from buffalo					**Subtotal**
		**Negative**	**0.3**	**0.6**	**0.9**	**1.2**	
*Antibody titers against BPIV3a SF strain	Negative	3	1	2	1		7
	0.3				1		1
	0.6		1	2	1	2	6
	0.9			3	4	1	8
	1.2					2	2
Subtotal		3	2	7	7	5	24

* Neutralization titers were calculated as the decimal logarithm of the reciprocal of the last dilution with clear cytopathic effects.

Each cell contains the number of animals with the antibody titers against each virus.

## Discussion

In the present study, we describe the isolation of BPIV3 from two water buffaloes with respiratory and reproductive signs. The virus was isolated from nasal and vaginal swabs from two out of twelve animals with reproductive and respiratory signs studied. To our knowledge little is known about the pathogenesis of this virus in buffalo. Should there be a syndrome similar to that present in cattle where the virus is transiently excreted [27], the unsuccessful virus isolation from the then remaining symptomatic animals could be related to the time point of sampling, which could have taken place at the end of the acute infection.

The IF assay showed strong and specific binding of anti-BPIV3 antibodies to cells infected with these three positive clinical isolates. Sera from buffaloes were able to neutralize the activity of the 7 N, 7 V and 2 V isolates and that of the reference bovine virus (BPIV3 strain SF), but the neutralization titers against the latter were lower than against buffalo isolates, suggesting antigenic differences.

The sequence of the M amplified fragment showed 94% nucleotide identity between buffalo isolates and BPIV3b strain Q5592 from Australia [8]. This is the first report of molecular characterization of a BPIV3 isolated from buffaloes. In order to determine homology between PIV3 isolates from Argentinean bovine and buffaloes, we characterized retrospectively six isolates collected from bovine respiratory outbreaks that occurred in 2009 and 2011. These isolates showed high percentages of nucleotide and amino acid identity with A and C genotypes (Table 1). There are only few reports about BPIV3 circulation in Argentina, Epstein *et al* described the isolation, virological and physicochemical characterization of PIV3 from Argentinean cattle and sheep in 1974 [23]. However, phylogenetic studies of these isolates were not carried out. Phylogenetic reconstructions based on the alignment of the M-gene nucleotide sequences demonstrated that the 7 N, 7 V and 2 V isolates clustered in the B genotype group. So far, this genotype has only been previously reported in Australia [8]. The phylogenetic position of the Argentinean buffalo isolates might be correlated with the antigenic differences observed by cross-neutralization studies, where the reference strain belonging to the BPIV3a group was lesser neutralized than the buffalo viral isolates by sera of buffaloes. Therefore the serological and molecular characterization of buffalo BPVI3 isolates show that these viruses are more similar to B than to A (SF reference strain) and C BPVI3 genotypes (SD0835 reference strain). Interestingly, the genotype B isolated from buffaloes was not found among the bovine samples, analyzed in this work, that clustered with genotypes A and C. Genotype B was until now observed only in Australia with the hypothesis of a recent crossing from another species into cattle [8]. This evidence of genotype B in buffaloes should need further investigation to know if this species could be one of the host species of this genotype. Therefore analysis of a larger number of isolates from cattle and buffaloes is needed to determine whether the three genotypes circulate in both species, or if the distribution observed is representative. Importantly, this is the first report of bovine B and C genotype circulation out of Australia and China, respectively. The circulation of genotype C only in China was attributed to a geographic restriction [7]. This hypothesis can be ruled out by our results and this observation was most likely due to the relative frequencies of these genotypes with respect to the samples analyzed.

To our knowledge this is the first report of the isolation of a parainfluenza virus type 3 from vaginal secretions. However, it is important to highlight that the presence of the virus in the secretions could be due to oro-genital contact between animals. Evidence of viral replication in the genital mucosa needs new investigations.

## Conclusion

The isolation and characterization of BPIV3 from water buffaloes were reported. We identified the isolates as bovine parainfluenza virus type 3 genotype B that differs from genotypes A and C circulating in Argentinean cattle herds reported so far. Further studies would be required to determine if A and C genotypes also circulate in buffalo herds, as well as to elucidate the role of buffalo as reservoirs, especially of the B genotype, and in the transmission of this virus to cattle.

## Methods

### Samples

Nasal and vaginal swabs were obtained from 12 water buffaloes of a dairy farm in the Argentinean province of Chaco, during an outbreak of respiratory and reproductive disease in July 2009. These animals had white stringy mucus in the nose and vulva. These samples were inoculated on Madin Darby bovine kidney (MDBK) cell cultures. The SF strain of BPIV3 was used as reference. Cells were daily examined under optical microscope to observe the appearance of cytopathic effects.

Bovine isolates were obtained from cattle with respiratory syndromes, 5 of them in the year 2009 and the remaining isolate in 2011. They were collected and amplified from Azul laboratories (Azul-Buenos Aires province) from outbreaks of Buenos Aires province.

### Immunofluorescence test

Virus isolates were inoculated into confluent MDBK cells in 8-well chambers at a MOI of 1.0, 0.1, and 0.01. For direct IF test, Cells with cytopathic effects were fixed with 99.5% acetone and then incubated with FITC anti-BPVI3 IgG (Ames). For indirect IF, cells were incubated with antibovine PIV3 polyclonal antibody (NVSL) diluted 1:100 in PBS at 37 °C in a moist environment for 1 h. Then, cells were incubated with FITC antibovine IgG (KPL). In both cases, slides were layered with buffered glycerin and observed under epifluorescence in an Olympus BX 40 + H hal microscope.

### Transmission electron microscopy

Twenty-five μl of viral suspension (16 HAU) were placed on a collodion membrane supported on a copper grid, negatively stained with phosphotungstic acid, and observed with a JEOL 1200 EX II transmission electron microscope [28].

### Hemagglutination assay (HA) and Haemagglutination inhibition assay (HIA)

Serial two-fold dilutions of culture supernatants from infected MDBK (50 ul) were titrated against 50 ul Guinea pig erythrocytes in V-bottom microtiter plates (Nunc). After incubation at 4°C for 4 h, hemagglutination was evaluated by the appearance or absence of a red cell button. Results were expressed as hemagglutinating units/50 μl (HAU/50 μl). The last dilution at which hemagglutination was observed was taken as the endpoint of the hemagglutinating activity. The reciprocal of this dilution expresses the number of hemagglutinating units or virus titer in UHA.

For the HI assay serial two-fold dilutions of sera from buffaloes were allowed to react with a fixed dose of reference viral haemagglutinin (8 UHA), followed by the addition of guinea pig agglutinable erythrocytes. Titers were expressed as the log10 of the reciprocal of the highest dilution hemagglutination inhibitory multiplied by a constant factor of 8.

### RT-PCR and sequencing

Infected MDBK cells were scraped off the plates and homogenized by three cycles of freezing and thawing. After an initial centrifugation at 3,000 g for 15 min, polyethylene glycol 8000 (Sigma), 10% (w/v, final concentration) was added to the cell lysates, followed by incubation for 4 h at 4°C. The virus was pelleted at 12,000 g for 60 min at 4°C. Viral genomic RNA was extracted from the virus pellet using RNeasy Mini Kit (QIAgen). Oligonucleotide primers for BPIV3 detection and identification (Mfwd: 5´AGTGATCTAGATGATGATCCA 3´ nt - 3960 and Mrev: 5´GTTATTGATCCAATTGCTGT −3´ nt - 4288) were designed based on a 328 bp segment of the consensus BPIV3 Matrix (M) gene. cDNA was synthesized using the specific oligonucleotide primer (Mfw) and MMLV reverse transcriptase (Promega). Subsequently, Go Taq DNA Polymerase (Promega) was used to amplify the M gene fragment.

Mfw and Mrev primers were tested for specificity by RT-PCR on BRSV (bovine respiratory syncytial virus) RNA, giving negative results. PCR products were purified using a Qiaquick PCR purification kit (Qiagen). Sequencing reactions were performed twice in both directions with BigDye Terminator v3.0 kit (Applied Biosystems, Lennik, Belgium) and analyzed with ABI Prism 3730 DNA Analyzer (Applied Biosystems).

### Phylogenetic analysis

M fragment nucleotide and predicted amino acid sequences of buffalo isolates were edited and analyzed with BioEdit version 7.0.5.3 [29]. Alignments of these sequences together with other from representative isolates of previously identified BPIV3 and human parainfluenza virus type 3 (HPIV3) were created by Clustal W. Nucleotide sequence alignments of the M gene fragment were used for phylogenetic analyses using MEGA software, version 4 [30]. Phylogenetic relationships were investigated by bootstrap analysis (1000 replicates) using the Neighbor joining method with the Kimura two-parameter model for nucleotide data analysis. GenBank accession numbers of the sequences used in the phylogenetic tree are as follows: BPIV3 reference strains: BPIV3 strain Kansas/15626/84, D84095; BPIV3 strain, NC 002161, AF178654; SF strain AF178655; Q5592, EU277658; SD0835, HQ530153; BPI3 JCU strain, EF 108221; BP4158 stain EF 108222; BP4159 strain, EF 108223; BP6121 strain, EF 108224; BP6128 strain, EF 108225; BP7393, EU266069; ISU92, EU439428; Texas-81, EU439429 and HPIV3 reference strains: HPIV strains, M16458, M16569; HPIV3-Gpv strain, NC 001796 and 14702 strain, EU424062. Sendai virus (AB005795) as outgroup.

### Serum neutralization test

Cross-neutralization tests were performed in MDBK 96-well plates, by overnight incubation of 200 TCID_50_ on 50 ul of 7 N buffalo virus isolates or BPIV3 reference strain (SF) with 50 ul heat-inactivated two-fold diluted sera. The plate was incubated for 5 days at 37 °C, under 5% CO2, and then screened for the presence or absence of cytopathic effects under optical microscope (400x magnification) to determine the virus-neutralization titers. Neutralization titers were calculated as the decimal logarithm of the reciprocal of the last serum dilution with clear cytopathic effects.

## Authors’ contributions

SM and SR designed the experiments, analyzed the data and drafted the manuscript together. SM performed the experiments. PL, GC, JK, and GC gently surrendered the field isolates. DR and VP kindly provided the reference strain. AM and ET participated in the molecular genetic studies, interpretation of data and contributed to the manuscript. MC helped to draft the manuscript. JD helped with electron microscopy. OZ helped with cell cultures. All authors read and approved the final manuscript.
